# Plant structural and storage glucans trigger distinct transcriptional responses that modulate the motility of *Xanthomonas* pathogens

**DOI:** 10.1128/spectrum.02280-23

**Published:** 2023-10-19

**Authors:** Isabela Mendes Bonfim, Douglas Alvarez Paixão, Maxuel de Oliveira Andrade, Joaquim Martins Junior, Gabriela Felix Persinoti, Priscila Oliveira de Giuseppe, Mário Tyago Murakami

**Affiliations:** 1 Brazilian Biorenewables National Laboratory (LNBR), Brazilian Center for Research in Energy and Materials (CNPEM), São Paulo, Brazil; 2 Graduate Program in Molecular and Morphofunctional Biology, Institute of Biology, University of Campinas, São Paulo, Brazil; CCG-UNAM, Cuernavaca, Mexico

**Keywords:** RNA-seq, transcriptome, regulation, *Xanthomonas*, phytopathogens, CAZyme, polysaccharide utilization loci, cellobiose, starch, glucans, motility

## Abstract

Some phytopathogens are outfitted with a broad and diverse repertoire of enzymatic systems that enable the breakdown and utilization of host polysaccharides as a source of carbon, energy, and stimuli. However, the functional assignment of these enzymatic systems and the influence of their products on modulating pathogen behavior during host colonization are yet poorly comprehended. In this study, we performed RNA-seq analyses to provide a comprehensive, genome-wide view of the transcriptional response of the model phytopathogen *Xanthomonas citri* pv. *citri* 306 (known as *X. citri* 306) to cellobiose, a component of structural β-1,4-glucans (majorly cellulose), and storage α-glucans, seeking to better understand how they are assimilated and the impacts of their sensing on bacterial behavior and physiology. Structural β-1,4-glucans and storage α-glucans (starch) are spatially discretized in the plant cell, therefore representing spatiotemporal references for the bacterium during host colonization. Combining transcriptional and genome mining analyses with gene knockout and cell motility assays, we show that *X. citri* 306 harbors molecular systems for the breakdown and assimilation of these carbohydrates, revealing that cellobiose upregulates genes related to flagellum assembly and type IV pili, inducing a higher motility state. In contrast, starch suppresses genes related to chemotaxis, flagellum assembly, and biofilm dispersion, decreasing motility. Taken together, these results unravel that, besides using structural β-glucans and storage α-glucans as sources of carbon and energy, *Xanthomonas* bacteria also sense them, adapting their metabolism and controlling transitions between higher and lower motility states for successful host colonization.

**IMPORTANCE:**

Pathogenic *Xanthomonas* bacteria can affect a variety of economically relevant crops causing losses in productivity, limiting commercialization and requiring phytosanitary measures. These plant pathogens exhibit high level of host and tissue specificity through multiple molecular strategies including several secretion systems, effector proteins, and a broad repertoire of carbohydrate-active enzymes (CAZymes). Many of these CAZymes act on the plant cell wall and storage carbohydrates, such as cellulose and starch, releasing products used as nutrients and modulators of transcriptional responses to support host colonization by mechanisms yet poorly understood. Here, we reveal that structural and storage β-glucans from the plant cell function as spatial markers, providing distinct chemical stimuli that modulate the transition between higher and lower motility states in *Xanthomonas citri*, a key virulence trait for many bacterial pathogens.

## INTRODUCTION

Carbohydrates are generally seen as a source of carbon and energy for living cells. However, their roles in life go far beyond that, as they trigger responses in the cells related not only to their uptake and metabolism but also to other physiological processes relevant to cell survival, adaptation, and growth (1). This has been clearly evidenced in pathogen–host interactions, with several studies showing that pathogens from either animals or plants exploit carbohydrates and other nutrients to regulate their virulence and adapt their metabolism for a successful colonization (2, 3).

For *Xanthomonas* phytopathogens, host carbohydrates serve as an important source of energy, carbon, and stimuli (4); however, in great part due to the sheer complexity of plant carbohydrates and associated enzymatic systems, the mechanisms governing these pivotal processes are still poorly understood. For instance, both cellulose and starch are composed exclusively of the same monosaccharide, glucose, but they display different structures and stereochemistry, leading to utterly distinct biophysical properties and functional roles. In cellulose, the glucose monomers are linked to each other via β-1,4-glycosidic linkages that result in long and linear polymers that tend to crystallize (5), whereas in starch, glucosyl residues are linked via α-1,4 or α-1,6 bonds, forming branched structures that are easier to depolymerize (6). Thus, cellulose provides mechanical strength to the plant cell wall, while starch is accumulated in intracellular organelles, providing a way to store energy and carbon in a dense and osmotically inert form that can be efficiently depolymerized to support plant metabolism and growth during the dark (7). Furthermore, based on their distinct location in the plant cell, these carbohydrates also might represent spatiotemporal references for pathogens during host infection and colonization.

Most pathogenic xanthomonads conserve genes encoding putative endo-β-1,4-glucanases and β-glucosidases as well as amylases and α-glucosidases, indicating their capacity to break down and utilize cellulose and starch (8). In particular, genes related to starch depolymerization usually colocalize with genes encoding for TonB-dependent transporters (TBDT) (9) and such genomic organization is known as carbohydrate utilization containing TBDT (10) systems in xanthomonads and as polysaccharide utilization loci (11) in the phylum *Bacteroidota*. Despite the genetic potential of *Xanthomonas* pathogens to cope with cellulose and starch, it has not yet been demonstrated how the sensing and assimilation of these carbohydrates affect its physiology and metabolism.

Therefore, in this work, we used *Xanthomonas citri* pv. *citri* 306 (known as *X. citri* 306), the causal agent of citrus canker, as a phytopathogen model to investigate, on a genome-wide scale, the molecular adaptations associated with the use of cellobiose, a major intermediate product of cellulose degradation, and starch as a source of carbon, energy, and signals to modulate the bacterial metabolism and behavior during host colonization. Remarkably, these carbohydrates, or better, the stereochemistry of their intermediate degradation products, α- or β-linked glucosyl units, oppositely modulate the bacterial motility, principally via flagellum-dependent mechanisms. Taken together, we propose that the sensing of cellobiose chemically indicates to the pathogen that the exterior of a host cell has been reached, whereas the sensing of storage α-glucans informs that the physical barrier imposed by the cell wall was overcome and the cytosol content was accessed, thus favoring the sessile phase. These results highlight the importance of these carbohydrates in adapting *Xanthomonas* metabolism and motility behavior to promote a successful host infection and colonization.

## RESULTS

### Unveiling the cellulose breakdown and uptake cascade in *X. citri* 306

**Fig 1:**
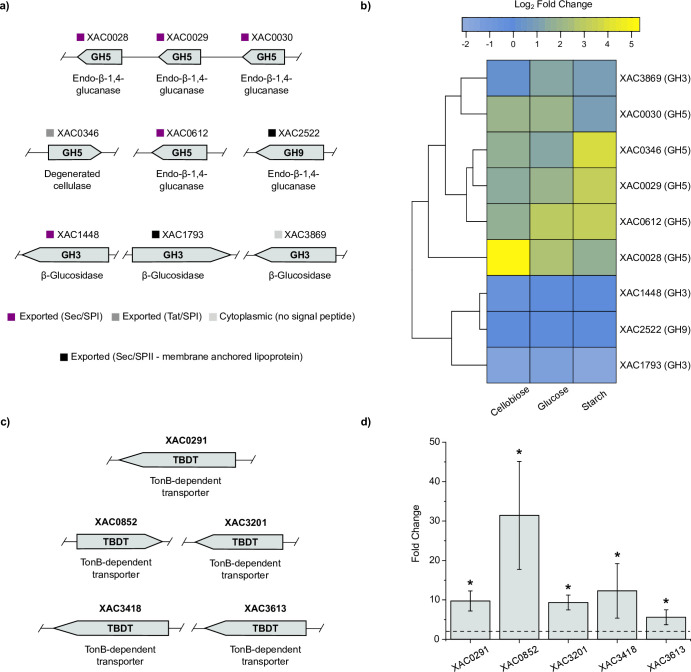
Cellulose utilization system in *X. citri* 306. (**a**) Endoglucanases related to β-1,4-glucan degradation in *X. citri* 306 genome. Signal peptide prediction (SignalP v.6.0 [14]) is indicated to each CAZyme. (**b**) Heatmap showing the transcription levels (log_2_ fold change) of endo-β-1,4-glucanase and β-glucosidase genes on RNA-seq experiments in different growth conditions compared to the LBON medium. (**c**) TBDT genes upregulated in the cellobiose condition compared to the glucose reference, as shown in panel (**d**). Transcription levels (fold change) are shown as mean ± SD from four biologically independent experiments (*n* = 4). Genes were considered differentially expressed (*) if *P*-adjusted <0.05 and fold change > 2.

The microbial depolymerization of cellulose commonly requires the action of endo-β-1,4-glucanases and/or cellobiohydrolases. The analysis of *X. citri* 306 genome revealed five proteins predicted as endo-β-1,4-glucanases from the family GH5 (XAC0028, XAC0029, XAC0030, XAC0346, and XAC0612), one from the family GH8 (XAC3516), and one from the family GH9 (XAC2522) (Fig. 1a). Yet related to glucanase activity, it is worth mentioning that *X. citri* 306 also contains a GH74 endo-β-1,4-glucanase; however, it is specific to xyloglucan (*Xac*Xeg74) (12). Of note, this strain is devoid of a GH6 cellobiohydrolase (cbsA) that is found in some xanthomonads colonizing the vascular tissues of plants (13).

To find clues about which genes could contribute with the breakdown of cellulose in *X. citri* 306, we analyzed the transcriptional response to cellobiose compared to the glucose and the rich medium conditions (Data Set S1), since this disaccharide is a major product intermediate of cellulose degradation cascade. Compared to the rich medium (LBON), the aforementioned genes XAC0028, XAC0030, XAC0346, and XAC0612 were upregulated in the presence of cellobiose, but some of them were also in the presence of glucose and/or starch, suggesting that the stimuli to trigger their expression are not exclusively dependent on the presence of cello-oligosaccharides in the medium (Fig. 1b; Data Set S1).

Despite the high adjusted *p*-value, XAC0029 displayed an average log_2_ fold change of 1.6 in the cellobiose condition compared to the rich medium, consistent with values found in the other tested conditions, except for that containing starch (log_2_ fold change, ~3.2) (Fig. 1b; Data Set S1). XAC0029 along with XAC0028, XAC0612, and XAC0346 belongs to the quorum sensing regulon mediated by diffusible signal factor (15), which adds another layer of complexity in the modulation of their expression. XAC0612 and XAC0028 have already been demonstrated to have endo-β-1,4-glucanase activity in *X. citri* 29-1 (16), but *in vitro* studies show that XAC0346 does not display endo-β-1,4-glucanase activity (data not shown). The biological role of XAC0029 and XAC0030 remains so far elusive, although the XAC0029 ortholog in *Xanthomonas oryzae* pv. *oryzae* (XOO0282; 88% sequence identity) was shown to have endo-β-1,4-glucanase activity and to be important for full virulence in rice (17).

For the GH8 (XAC3516) and GH9 (XAC2522) encoding genes, no activation was observed (Data Set S1). The genomic context of GH8 gene indicates a role for this characterized endo-β-1,4-glucanase (18) during bacterial cellulose synthesis, likely cleaving the nascent chains for their release to the extracellular medium. For the GH9 enzyme, it is still elusive its importance for cellulose depolymerization in xanthomonads, but the endo-β-1,4-glucanase activity of the ortholog XCC2387 (80% sequence identity) has been previously shown (19).

To further understand the uptake of cellobiose, we compared the expression of TBDTs between the cellobiose and the glucose conditions (Data Set S1). It revealed the activation of five putative TBDTs (XAC0291, XAC0852, XAC3201, XAC3418, and XAC3613), which could be involved with cello-oligosaccharides uptake to the periplasm (Fig. 1c and d). When comparing the expression of these TBDT genes in the other growth conditions, we noticed that they were significantly activated only in the presence of cellobiose, besides being already reported to be also activated in the presence of xyloglucan oligosaccharides (12), further supporting that cello-oligosaccharides are inducers of their expression and suggesting either a role for these TBDTs in cello-oligosaccharides uptake or a signaling cross-talk to activate other processes dependent on these TBDTs in *X. citri* 306.

Regarding the periplasmatic monomerization of cello-oligosaccharides, *X. citri* 306 is devoid of GH1 β-glucosidases but contains three GH3 enzymes previously shown to play a role in cello-oligosaccharides hydrolysis (XAC1448, XAC1793, and XAC3869) (12). In comparison to rich medium LBON, XAC3869 was the only one presenting a positive fold change in the presence of cellobiose, suggesting a major role in cellobiose hydrolysis (Fig. 1b: Data Set S1).

These genomic and transcriptomics analyses allowed to propose the biochemical steps involved in cellulose depolymerization and uptake in *X. citri* 306, indicating that, despite this species is not considered to have an efficient machinery to break down the plant cell wall, it contains canonical enzymes and TBDTs for cellulose utilization.

### Deciphering the starch utilization machinery in *X. citri* 306

**Fig 2:**
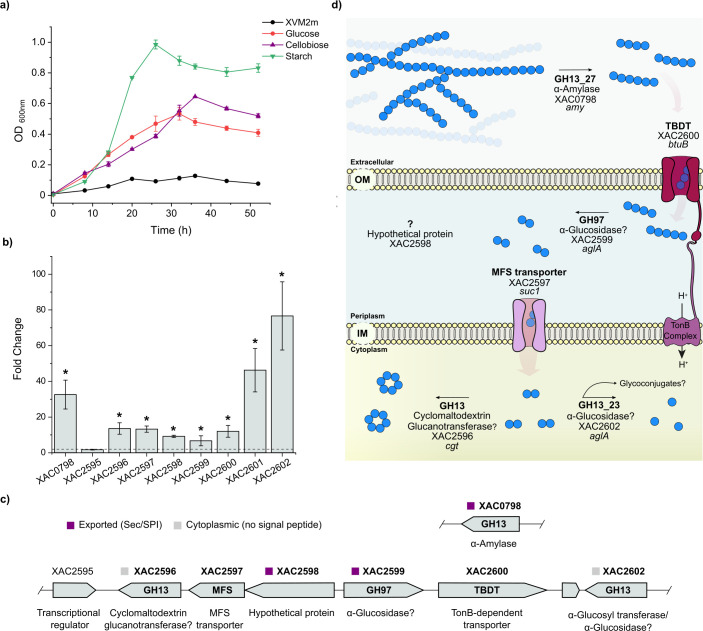
Starch utilization system of *X. citri* 306. Growth curves analysis of *X. citri* 306 in minimal medium XMV2m with different carbohydrate sources. Data are presented as mean ± SD from three independent experiments (*n* = 3). (**b**) Transcription level (fold change) of starch utilization genes based on RNA-seq data from the starch condition compared to the glucose condition. Data are shown as mean ± SD from four biologically independent experiments (*n* = 4). Genes were considered differentially expressed (*) if *P*-adjusted <0.05 and fold change > 2. (**c**) Predicted starch utilization system (Sus) in *X. citri* 306 genome, showing a LacI-family transcriptional regulator, MFS and TBDT transporters, glycoside hydrolases, and hypothetical proteins. The hypothetical protein XAC2598 has 780 residues and displays a short motif (163-342) homologous to amylo-α-1,6-glucosidases (22) but has not been assigned to a known GH family yet. Differentially expressed genes are highlighted in boldface. Signal peptide prediction (SignalP v.6.0 [14]) is indicated to each CAZyme. Biochemical activities with a question mark require experimental validation. (**d**) Putative model of how starch is progressively depolymerized by *X. citri* 306 enzymes and internalized by transmembrane transporters. Blue circles represent glucose residues, and the arrows represent the enzymes. OM: outer membrane; IM: inner membrane.

Interestingly, *X. citri* 306 displays a superior growth on starch compared to glucose or cellobiose, indicating that it has an efficient enzymatic machinery for the utilization of this polysaccharide (Fig. 2a). The *X. citri* 306 genome harbors several genes potentially involved with starch depolymerization including 1–3 belonging to the families GH4, GH15, and GH97 and notably 12 belonging to the family GH13 (Table S1). However, only some members of the families GH13 (XAC0798, XAC2596, and XAC2602) and GH97 (XAC2599) were upregulated in the starch condition (Fig. 2b; Data Set S1), indicating that they play a role in starch depolymerization. Some of them are next to the loci encoding for a transcriptional regulator (XAC2595), an inner membrane MFS transporter (XAC2597), and a TonB-dependent transporter (TBDT, XAC2600), with the last two upregulated in presence of starch (Fig. 2b and c). Of note, XAC2595 is a transcriptional regulator belonging to the LacI family, implying that it might repress the expression of XAC0798 and the loci XAC2596-XAC2602 in the absence of starch depolymerization products. This integrated genomic and transcriptomic analysis indicates that the gene cluster XAC2595-XAC2602 along with the gene XAC0798, which encodes the α-amylase Amy (20), composes the starch utilization system of *X. citri* 306 (Fig. 2c). Indeed, the disruption of the *amy* gene impairs cells to degrade starch, although without affecting the pathogenicity of *X. citri* 306 (21) but reducing the virulence of *Xanthomonas campestris* in radish plants (20).

Based on our results, literature data, and subcellular localization prediction analysis (Table S2), we propose a putative enzymatic cascade for starch depolymerization in *X. citri* 306 (Fig. 2d). It starts with an extracellular α-amylase (XAC0798; GH13_27 subfamily) that catalyzes the endo-hydrolysis of α-1,4-ᴅ-glucosidic linkages in starch, similarly to its homologous in *X. campestris pv. campestris* strain 8004 (20, 23), releasing malto-oligosaccharides. Next, the malto-oligosaccharides are transported through the TBDT encoded by *btuB* (XAC2600) to the periplasm. Interestingly, besides XAC2600, other TBDT-encoding genes *iroN* (XAC3311) and *btuB* (XAC3444) had the expression activated in the starch condition (Data Set S1), suggesting that either more than one transporter could be related to starch oligosaccharides uptake or that starch also induces the uptake of other substrates. The genomic context of XAC3311 supports a role in carbohydrate uptake, due to its proximity to GH-encoding genes, but the same was not observed for *btuB* (XAC3444).

At the periplasm, the putative α-glucosidase encoded by XAC2599 (GH97) might hydrolyze α-glycosidic linkages, probably releasing maltose from the oligosaccharides. Although also predicted as periplasmic, the role of the hypothetical protein XAC2598 in this system remains to be determined. The mono and small oligosaccharides generated in the periplasm, such as maltose, are probably transported to the cytoplasm through the MFS transporter encoded by *suc1* (XAC2597). Next, the putative cyclomaltodextrin glucanotransferase XAC2596 (GH13, not yet assigned to a subfamily) likely catalyzes the chemical reaction of cyclizing the malto-oligosaccharides into cyclodextrins. The disaccharide maltose is probably cleaved by XAC2602, which shows high level of sequence identity (91%) with the α-glucosyl transferase XgtA from *X. campestris* WU-9701 (24). XgtA shows both α-glucosidase activity, with high level of substrate specificity to maltose, and α-glycosylation activity toward alcoholic and phenolic –OH groups, using maltose as an α-glucosyl donor to generate glycoconjugates *in vitro* (25). The physiological relevance of this α-glycosylation activity is still elusive, but a possible role for these glycoconjugates could be in signaling pathways since they seem to be specifically produced during starch processing in *X. citri* 306.

Taken together, these analyses indicate that key enzymatic activities for the complete depolymerization and uptake of starch are present in *X. citri* 306, corroborating the efficient utilization of starch as carbon and energy source.

### The antagonistic effect of cellobiose and starch on gene expression related to bacterial motility

**Fig 3:**
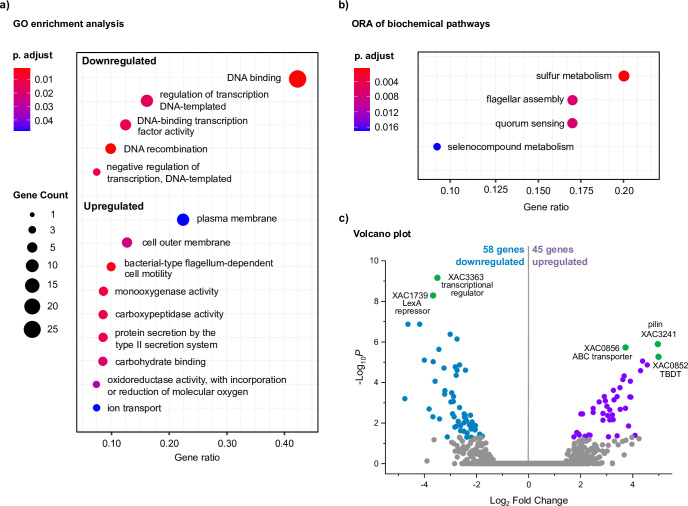
Transcriptional responses of *X. citri* 306 to cellobiose. (**a**) Gene ontology (GO) enrichment analysis and (b) over representation analysis (ORA) of biochemical pathways considering the differentially expressed genes (DEGs) in the minimal medium XVM2m containing cellobiose in comparison to the medium XVM2m containing glucose. Circles size and color represent the counts and BH-adjusted *p*-values, respectively. Gene ratio corresponds to the number of DEGs related to a GO term divided by the total number of annotated DEGs. (**c**) Volcano plot of RNA-seq data showing the DEGs in the presence of cellobiose. In green are indicated some of the DEGs with the highest values of −log_10_ P and | log_2_ fold change |. Thresholds: *P*-adjusted <0.05 and | log_2_ fold change | > 1.

Gene ontology (GO) enrichment analysis showed the activation of nine “biochemical pathway” categories in the presence of cellobiose, while other five were suppressed (Fig. 3a; Data Set S2). Most of the downregulated genes in the cellobiose condition are associated with DNA binding and recombination, or transcriptional regulation. Among them, three LysR regulators, abundant in the prokaryotic kingdom and typically related to virulence, metabolism, and quorum sensing (26), were exclusively downregulated in the cellobiose condition (XAC0255, XAC3459, and XAC2718), indicating that they might play a role in the regulation of cellobiose-specific responses. Among the upregulated genes, we would like to highlight the genes from the category “Bacterial-type flagellum-dependent cell motility” including *fliL* (XAC1948), *flgL* (XAC1976), *flgG* (XAC1981), and *flgE* (XAC1983), suggesting an increase in bacterial motility in the presence of cellobiose.

**Fig 4:**
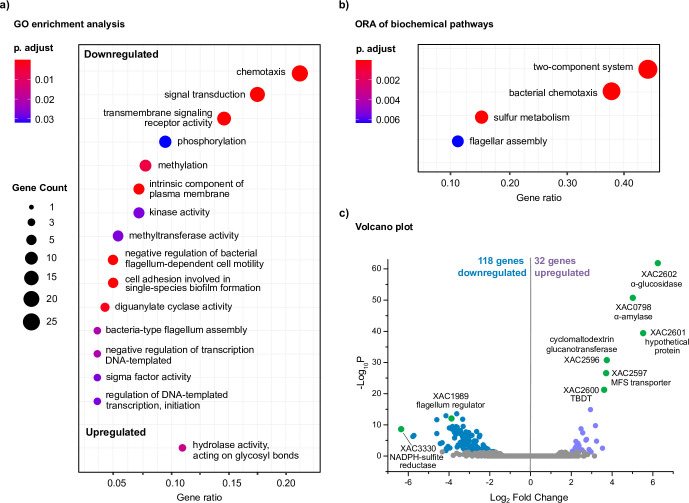
Transcriptional profile of *X. citri* 306 in the presence of starch in minimal medium. (**a**) Gene ontology (GO) enrichment analysis and (b) over representation analysis (ORA) of biochemical pathways considering the differentially expressed genes (DEGs) in the minimal medium XVM2m containing starch in comparison to the medium XVM2m containing glucose. Circles size and color represent the counts and BH-adjusted *p*-values, respectively. Gene ratio corresponds to the number of DEGs related to a GO term divided by the total number of annotated DEGs. (**c**) Volcano plot of RNA-seq data showing the DEGs in the presence of starch. In green are indicated some of the DEGs with the highest values of −log_10_ P and | log_2_ fold change |. Thresholds: *P*-adjusted <0.05 and | log_2_ fold change | > 1.

Remarkably, in the starch condition, most GO categories were suppressed (15 in total) and only one was activated, “Hydrolase activity on glycosyl bonds” (Fig. 4a). Genes from the upregulated GO category include three Sus genes, which are between those with the highest values of −log_10_ P and log_2_ fold change (Fig. 4c), indicating that the focus of *X. citri* 306 when exposed to starch is the breakdown and uptake of this carbohydrate. Regarding the 15 downregulated GO categories in the starch condition, we highlight those involved in the sensing of intracellular or environmental signals and those related to flagellum-dependent cell motility (27) (Data Set S2).

Interestingly, the transcriptional regulators *flgM* (XAC1989, anti-σ factor) and *fliA* (XAC1933, σ factor) (28) were repressed by starch, which correlates with the downregulation of flagellum-related genes, including those encoding for the chaperones FliS (XAC1973)*,* FlgA (XAC1988), and FlgN (XAC1990) (29 – 31). *fliA* and *flgM* expression has been demonstrated to be activated by the transcription factor XbmR (XAC3733) (32) and the deletion of *xbmR* in *X. citri* 306 was shown to impair chemotaxis and motility via the downregulation of *fliA* and *flgM*, similar to the transcriptional response triggered by starch. However, *xbmR* expression was not downregulated by starch, leading us to hypothesize that starch-derived products might somehow inhibit XbmR function at the posttranslational level, thus decreasing the expression of chemotaxis, flagellar assembly, and biofilm dispersion genes via FliA- and FlgM-dependent mechanisms.

Over representation analysis (ORA) of biochemical pathways also highlighted that the category “flagellar assembly” is overrepresented in both cellobiose and starch conditions (Fig. 3b and 4b; Tables S3 and S4; Data Set S1); however, they have antagonistic effect, since cellobiose triggers responses favoring cell motility whereas starch suppresses it via the modulation of distinct subsets of genes. Other oppositely regulated biochemical pathway highlighted in ORA analyses is for sulfur metabolism, but the biological meaning of this distinct response remains to be determined (Fig. 3b and 4b; Data Set S1; Table S3 and Table S4).

Taken together, these results indicate that the sensing of these carbohydrates, representing distinct subcellular environments in the host, might be a relevant mechanism controlling bacterial motility, an essential virulence trait for many plant pathogens.

### Cellobiose and starch oppositely modulate the swimming motility in *X. citri* 306

As described in the previous sections, cellobiose and starch triggered opposite transcriptional responses related to flagellum-dependent cell motility in *X. citri* 306. However, there are at least three different sorts of cellular motilities described for *X. citri* 306 so far including (i) swimming motility, which is dependent on flagella; (ii) twitching motility, which is dependent on the type IV pilus but not on flagella; and sliding motility, which is also a type of flagellum-independent motility involving xanthan gum as a surfactant (33 – 35).

To better investigate the signaling influence of cellobiose and starch in bacterial swimming motility, we performed an in-plate assay in minimal medium containing low agar concentration supplemented with either starch or cellobiose and measured the bacterial cell spreading of WT *X. citri* 306 and the knockout for *fliC,* which encodes the flagellin subunits essential for flagellum assembly. To evaluate eventual contributions of other types of motilities to the phenotype observed in the swimming test, we also tested individual knockouts that have been reported as essential for twitching motility XAC2924 (*pilT*) (35) and sliding motility (*gumD*) (33). As a negative control, we assayed the motility of individual XAC2868 (*vieA*) and XAC2870 (*cheB*) gene knockouts, which are genes related with chemotaxis regulation but that were not differentially expressed in the starch or cellobiose conditions.

**Fig 5:**
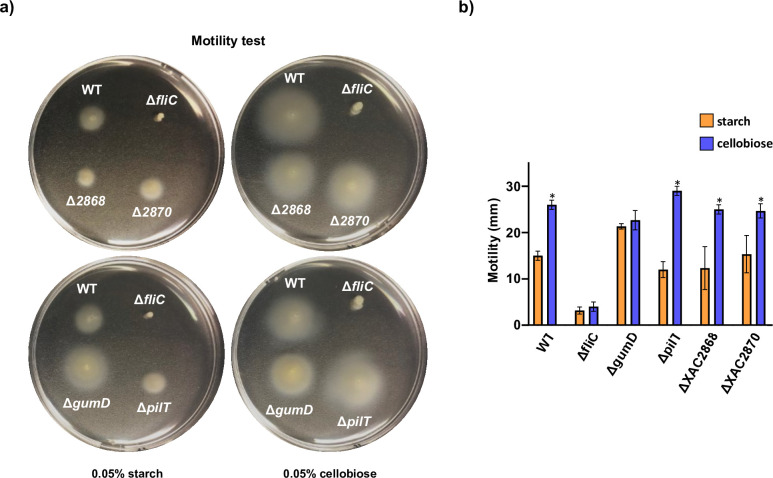
Swimming motility assays performed on soft agar showing that cellobiose and starch have opposite effect on regulation of *X. citri* 306 flagella-dependent motility. (**a**) Wild type (WT) and the mutant strains *ΔpilT*, *ΔXAC2868*, and *ΔXAC2870* significantly increased their motility on cellobiose, while *ΔgumD* mutant strain showed similar spread on both conditions. Also, deletion of *fliC* impaired the swimming motility on tested conditions. Cells were spotted and experiments were analysed after 72 h of incubation at 28°C. (**b**) Measurements of the diameters of cell spread by swimming motility on cellobiose and starch plates after 72 h of incubation at 28°C. The means of three biological replicates and standard deviation values are shown. Asterisk indicates significant difference based on one-way ANOVA with Tukey’s test (*P* < 0.001) compared with the starch condition.

As expected, WT swimming motility in the cellobiose condition was significantly higher compared to that on the starch condition (Fig. 5). Δ*fliC* mutation impaired motility on both assayed conditions, indicating that the motility difference observed for the WT strain in the cellobiose and starch conditions was flagellar dependent, *i.e.*, a swimming motility. Δ*pilT*, ΔXAC2868, and ΔXAC2870 strains showed a WT-like phenotype, in agreement with our transcriptome data showing that these genes were not differentially expressed in the cellobiose and starch conditions (Fig. 5). Interestingly, the swimming motility of the Δ*gumD* mutant, which is deficient in xanthan gum production, was higher compared to that of the WT in the starch condition. Thus, the reduction in xanthan gum production might facilitate the swimming motility in *X. citri* 306*,* which has been previously reported for other exopolysaccharides in bacteria (36). These *in vivo* results indicate that cellobiose and starch (or products derived from their depolymerization) act as opposite signals that modulate swimming motility by reprograming the gene expression in *X. citri* 306.

## DISCUSSION

Signaling molecules are primarily responsible for dictating behavioral responses in bacteria, either by directing their movements across the environment, indicating the need to activate protective mechanisms against stress or even by showing them the possibility of conquering new niches. Similarly to what occurs with animal pathogenic bacteria (37), here we show that host-cell-derived carbohydrates also function as spatial guiders for phytopathogenic bacteria, indicating where they are within the host and stimulating appropriate responses to each niche experienced during the infection process.

The presence of a *X. citri* pv. *citri* strain within damaged plant cells has been observed for the virulent strain T in a previous work (38) and transcriptional activation of the *amy* gene has been reported in *X. campestris* pv. *campestris* at the end of the biotrophic phase of plant infection (39), supporting the hypothesis that these bacteria might have access to the cytoplasmic content and consequently to the starch granules during the host colonization. When *X. citri* 306 is exposed to starch, motility and biofilm dispersion are inhibited, favoring cell growth as supported by comparative growth analysis in the carbohydrates evaluated in this study (Fig. 2a). This higher-to-lower mobility state transition correlated with changes in the expression of genes related to sulfur metabolism, with cellobiose triggering sulfur uptake and assimilation and starch repressing it via the modulation of a distinct subsets of genes (Data Sets S1 and S2). Association between sulfur assimilation and flagellar function have been observed in the pathogen *Serratia marcescens* (40), for example, but it remains elusive in *Xanthomonas* species.

To use complex plant polysaccharides as sources of carbon, energy, and stimuli, *X. citri* 306 has diverse gene sets dedicated to depolymerizing, uptake, and metabolizing these biopolymers. Based on our results, we revealed previously unidentified loci for starch utilization (XAC2595-XAC2602) and highlighted some knowledge gaps in the biochemical characterization of *Xanthomonas* spp. endo-β-1,4-glucanases, which may guide future studies aiming at a deeper understanding of β-glucan utilization systems in this bacterial genus. Notably, we observed the activation of some TBDTs by more than one type of carbohydrate, suggesting that they might play a role in the uptake of oligosaccharides from different sources.

To the best of our knowledge, this study is the first to show that a phytopathogenic bacterium can recognize stereochemical differences between cellobiose (β-glucan) and starch (α-glucan), two glucose-based carbohydrates, triggering distinct genome-wide transcriptional responses, which have antagonistic effect regarding bacterial motility. As motility has been considered a key virulence trait for many bacterial pathogens, this work unveils a novel molecular mechanism, based on the stereochemistry of glycosyl linkages, for the modulation of this essential function for a successful host colonization. Furthermore, this work highlights a fundamental aspect of plant pathogens in how they have been co-evolving to use, sense, and adapt their responses to the chemical and structural diversity of plant polysaccharides.

## MATERIALS AND METHODS

### Bacterial strains, plasmids, and growth curves analysis

Strains and plasmids used in this study are listed in Table S5. *Escherichia coli* cells were cultured in Lysogeny broth (LB, 10 g⋅L^−1^ tryptone, 5 g⋅L^−1^ yeast extract, and 10 g⋅L^−1^ NaCl, pH 7) medium or LB agar plates at 37°C. *X. citri* 306 strains were cultured in LBON medium (1% [mass/vol] bacto peptone and 0.5% [mass/vol yeast extract) and on LBON agar plates at 28°C. When required, media were supplemented with antibiotics: ampicillin (100 µg/mL) or kanamycin (50 µg/mL).

For growth curve analysis, *X. citri* 306 strain was cultured in LBON medium (1% [mass/vol] bacto peptone and 0.5% [mass/vol] yeast extract) containing 100 µg⋅mL^−1^ ampicillin at 30°C and 200 rpm until mid-exponential phase. Then, the cultures were centrifuged for 5 min at 6,000*g* and the harvested cells were washed once with the modified minimal medium XVM2 (41) (XVM2m, without sucrose and fructose) supplemented with glucose, cellobiose, or starch (5 mg⋅.mL^−1^) and transferred to a 20mL culture medium for an initial OD_600_ = 0.01. Growth analyses were performed at 30°C and 200 rpm, being monitored through OD_600_ readings at times 0 h, 8 h, 14 h, 20 h, 26 h, 32 h, 36 h, 44 h, and 52 h in triplicate.

### RNA extraction and cDNA library preparation

Total RNA samples were extracted from 15 mL *X*. *citri* 306 cultures grew at mid-exponential phase (as described above) using the TRIzol/chloroform protocol (42). Genomic DNA was removed by treatment with DnaseI (Invitrogen) and then then the samples were treated with RNaseOUT (Invitrogen), followed by purification with the Rneasy Mini Kit (Qiagen), according to the manufacturer’s recommendations. The absorbance analyzes were performed in a NanoDrop spectrophotometer (Thermo Scientific) and the integrity of the samples was evaluated in the Agilent 2100 Bioanalyzer (Agilent Technologies). Prior to the tests, the samples were quantified on a Qubit 2.0 Fluorometer using the RNA BR assay kit (Life Technologies).

For cDNA library preparation, only RNA samples free of genomic DNA contamination and with values of RIN (RNA Integrity Number) greater than 7 were used. Then, 2–2.5 µg of RNA was used for depletion of rRNA using the Ribo-Zero rRNA Removal Kit (Gram-Negative Bacteria, Epicenter Biotechnologies). The preparation of the cDNA libraries was performed using the TruSeq Stranded mRNA kit (Illumina Inc.) according to the manufacturer’s protocol. The quality of the samples was accessed using an Agilent 2100 Bioanalyzer (Agilent Technologies) and libraries were quantified via qPCR using the KAPA Library Quantification Kit (Illumina). Paired-end sequencing (100 bp) of the constructed libraries was carried out on an Illumina Hiseq 2500 system at LNBR (CNPEM, Campinas, Brazil).

### RNA sequencing and analysis

The raw sequencing reads were processed to remove the low-quality sequences and adapters, then filtered for removal of ribosomal RNA using the software SorteMeRNA 2.1 (43). The sequences were then mapped to the *X. citri* 306 genome (44) using the Bowtie2 program (45). The Rsubread software was then used to count reads mapped to *X. citri* 306 transcripts (46) (Table S6). All reads signed to coding genes were used for differential expression analysis by calculating the TPM values (transcribed per million reads) with the R DESeq2 package version 1.18.1 (47). The resulting values were log_2_ transformed and *t*-test was performed on these expression values to compare differential gene expression of *X. citri* 306 under the growth condition XVM2m containing different carbohydrates in comparison to XVM2m containing glucose or LBON medium.

Genes were considered differentially expressed according to Wald test and *p*-values were adjusted for multiple tests using the Benjamini-Hochberg (BH) method implemented in DESeq2 package, using │log_2_ fold change│≥1 and a *p-*adjusted ≤0.05 as a threshold. Analyzes of variance using the principal component analysis (PCA) method and hierarchical clustering based on the correlation of Pearson were also performed to examine data quality and comparability. Non-concordant replicas were removed from the analyses and disregarded to determine differentially expressed genes (DEG).

The Carbohydrate-Active enZymes Database (CAZy) (48) was used as a reference for the identification of the DEGs classified in CAZy families. The GO enrichment analysis was performed using the clusterProfiler 3.14.3R/Bioconductor package (49) and the categories were considered enriched based on hypergeometric test, implemented in the enrich function of the package. ORA of biochemical pathways was also performed using the clusterProfiler 3.14.3R/Bioconductor package (49). RNA-seq data for the glucose conditions have been previously deposited in the NCBI database under accession number PRJNA668298.

### Deletion of motility-associated genes in *X. citri* 306

Oligonucleotides used are listed in the Table S7. Restriction, digestion, and DNA ligation were performed in accordance with the manufacturer’s instructions (New England Biolabs, Ipswich, USA). To construct the Δ*fliC, ΔpilT, ΔgumD, ΔXAC2868*, and *ΔXAC2870* in-frame deletion mutants, we amplified approximately 1 kb of the upstream and downstream regions of the target genes by PCR from *X. citri* 306 genomic DNA, and the two fragments were fused by PCR to produce an in-frame deletion (50). The generated fragment was then cloned into the *HindIII* restriction site of the pNPTS138 suicide vector, generating the constructs for knocking-out the five listed motility-associated genes (Table S6). The construct was introduced into *X. citri* 306 cells by electroporation, and the target genes on wild-type strain were replaced by the deleted version after two recombination events as described previously (50, 51). The generated deletion mutants were confirmed by PCR.

### Motility assay

For motility assay of *X. citri* 306 strains on soft agar, bacteria cells grown in LBON media for 48 h at 28°C were stabbed in 0.25% (wt/vol) agar XVM2 modified medium plates containing 0.05% (wt/vol) cellobiose or starch instead of fructose and sucrose. Motility was evaluated and photographed after 3 d of incubation at 28°C. The assay was repeated twice times independently in triplicates with similar results.

## Data Availability

The RNA-seq data generated during the current study are available at the Gene Expression Omnibus (GEO) repository under the accession code GSE234477. Additional data that support the findings of this study are available from the corresponding authors on reasonable request.

## SUPPLEMENTAL MATERIAL

Data Set S1 spectrum.02280-23-s0001.xlsx List of differentially expressed genes on the presence of cellobiose or starch.

Data Set S2 spectrum.02280-23-s0002.xlsx GO enrichment analysis.

Supplemental tables spectrum.02280-23-s0003.docx Tables S1 to S7.

ASM does not own the copyrights to Supplemental Material that may be linked to, or accessed through, an article. The authors have granted ASM a non-exclusive, world-wide license to publish the Supplemental Material files. Please contact the corresponding author directly for reuse.
