# Supplementary material for: Plant structural and storage glucans trigger distinct transcriptional responses that modulate the motility of Xanthomonas pathogens
Source: Microbiol Spectr. 2023 Oct 19;11(6):e02280-23. doi: 10.1128/spectrum.02280-23 (PMC10714752; doi:10.1128/spectrum.02280-23)
Supplement: Supplemental tables — Tables S1 to S7. [file spectrum.02280-23-s0003.docx]

**Supplementary Table 1. List of *X. citri* 306 genes encoding glycoside hydrolases belonging to families usually associated with α-glucans depolymerization.** ^a^ Biochemical function characterized in *X. citri* or *Xanthomonas* sp. orthologs as reported in the references. ^b^Putative function based on the description available on KEGG (1) or Uniprot databases (2).

| **ID** | **Description** | **CAZy** | **Biochemical funcion^a,b^** | **Reference** |
| --- | --- | --- | --- | --- |
| XAC3081 | 6-phospho-β-glucosidase | GH4 | Hydrolysis of 6-phospho-β-D-glucosyl-(1→4)-D-glucose in D-glucose and D-glucose-6-phosphate^b^ | - |
| XAC0154 | maltosyltransferase | GH13_3 | Uses maltose-1-phosphate as the sugar donor to elongate linear or branched α-1,4-glucans^b^ | - |
| XAC0155 | trehalose synthase | GH13_16 | Generates trehalose from maltose^b^ | - |
| XAC0156 | 1,4-α-glucan branching enzyme | CBM48, GH13_9 | Catalyzes the formation of the α-1,6-D-glucosidic linkages in glycogen by scission of an α-1,4-linked oligosaccharide from growing glucan chains^b^ | - |
| XAC0426 | 1,4-α-glucan branching enzyme | CBM48, GH13_9 | Catalyzes the formation of the α-1,6-glucosidic linkages in glycogen by scission of an α-1,4-linked oligosaccharide from growing glucan chains^b^ | - |
| XAC0427 | maltooligosyltrehalose trehalohydrolase | CBM48, GH13_10 | Hydrolysis of α-1,4-D-glucosidic linkage in 4-α-D-[(α-1,4-glucanosyl)] _n_ trehalose to yield trehalose and α-(1-4)-D-glucan^b^ | - |
| XAC0429 | maltooligosyl trehalose synthase | GH13_26 | Catalyzes the conversion of maltooligosaccharide into the non-reducing saccharide, maltooligosyl trehalose (α-maltooligosyl α-D-glucoside) by intramolecular transglycosylation. ^b^ | - |
| XAC0431 | glycogen debranching enzyme | CBM48, GH13_11 | Debranching enzyme for glycogen catabolism^b^ | - |
| XAC0798 | α-amylase | GH13_27 | Endo-hydrolysis of α-1,4-D-glucosidic linkages in polysaccharides containing three or more α-1,4-linked D-glucose units^a^ | (3) |
| XAC2596 | cyclomaltodextrin glucanotransferase | GH13 | Catalyzes the reaction of cyclizing part of a 1,4-α-D-glucan molecule, generating non-reducing cyclic dextrins or cyclodextrins^b^ | - |
| XAC2602 | α-glucosidase/  α-glucosyl transferase | GH13_23 | Hydrolysis of glucosidic linkages in maltose or α-glycosylation activity toward alcoholic and phenolic –OH groups, using maltose as an α-glucosyl donor^a^ | (4,5) |

**Supplementary Table 1. Continued**.

| **ID** | **Description** | **CAZy** | **Biochemical funcion^a,b^** | **Reference** |
| --- | --- | --- | --- | --- |
| XAC3254 | glycogen debranching enzyme | CBM48, GH13_11 | Debranching enzyme for glycogen catabolism^b^ | - |
| XAC3490 | sucrose hydrolase | GH13_4 | Sucrose hydrolysis producing glucose and fructose^b^ | - |
| XAC1177 | hypothetical protein | GH15 | Not assigned | - |
| XAC3210 | hypothetical protein | GH15 | Not assigned | - |
| XAC4082 | hypothetical protein | GH15 | Not assigned | - |
| XAC2599 | α-glucosidase | GH97 | Exo-hydrolysis of α-glucosidic linkages in oligos or polysaccharides with the release of glucose^b^ | - |
| XAC3313 | α-glucosidase | GH97 | Exo-hydrolysis of α-glucosidic linkages in oligos or polysaccharides with the release of glucose^b^ | - |

**Supplementary Table 2. Sequence-based prediction of subcellular localization of proteins possibly related to starch depolymerization in *X. citri* 306.** The position of the start codon originally annotated for these ORFs was revised based on the reference sequences whose accession codes are shown in NR ID. TMH = transmembrane helix. CYT = cytoplasm, EXT = extracellular, PER = periplasm, OM = outer membrane.

| *Locus* | NR ID | Description | SignalP 6.0 (6) | TMHMM (7) | SOSUI/GramN (8) | Consensus | Criterium |
| --- | --- | --- | --- | --- | --- | --- | --- |
| XAC0798 | WP_015462876.1 | α-amylase | Sec/SPI  (1-14) | **-** | EXT | EXT | SPI site accessible |
| XAC2596 | WP_011051695.1 | cyclomaltodextrin glucanotransferase | Other | - | CYT | CYT | Lack of signal peptide |
| XAC2598 | WP_011051697.1 | conserved hypothetical protein | Sec/SPI  (1-21) | - | PER | PER | SPI site accessible/PER |
| XAC2599 | WP_011051698.1 | α-glucosidase | Sec/SPI  (1-35) | - | PER | PER | SPI site accessible/PER |
| XAC2602 | WP_011051701.1 | α-glucosidase | - | - | CYT | CYT | Lack of signal peptide |

**Supplementary Table 3.** Over Representation Analysis (ORA) of biochemical pathways for the cellobiose condition using glucose as reference.

| **ID** | **Description** | **Gene Ratio** | **Bg Ratio** | ***p*-value** | ***p*-adjust** | **q-value** | **Gene ID** | **Count** |
| --- | --- | --- | --- | --- | --- | --- | --- | --- |
| **xac00920** | Sulfur metabolism | 7/35 | 31/1340 | 7.50e-06 | 0.0002 | 0.0002 | XAC3329/XAC3328/XAC1018/XAC3197/XAC1019/XAC1017/XAC0334 | 7 |
| **xac02040** | Flagellar assembly | 6/35 | 41/1340 | 0.0005 | 0.006 | 0.006 | XAC1981/XAC1948/XAC1976/XAC1983/XAC1977/XAC1984 | 6 |
| **xac02024** | Quorum sensing | 6/35 | 46/1340 | 0.0009 | 0.009 | 0.007 | XAC0857/XAC0856/XAC0860/XAC0858/XAC2512/XAC0545 | 6 |
| **xac00450** | Selenocompound metabolism | 3/35 | 11/1340 | 0.002 | 0.02 | 0.014 | XAC3329/XAC3328/XAC0336 | 3 |

**Supplementary Table 4.** Over Representation Analysis (ORA) of biochemical pathways for the starch condition using glucose as reference.

| **ID** | **Description** | **Gene Ratio** | **Bg Ratio** | ***p*-value** | ***p*-adjust** | **q-value** | **Gene ID** | **Count** |
| --- | --- | --- | --- | --- | --- | --- | --- | --- |
| **xac02030** | Bacterial chemotaxis | 27/70 | 48/1340 | 1.08e-24 | 3.24e-23 | 2.62e-23 | XAC1893/XAC1902/XAC1666/XAC1908/XAC1891/XAC1746/XAC2447/XAC1899/XAC0611/XAC1906/XAC1930/XAC1897/XAC1896/XAC1903/XAC1996/XAC2448/XAC1932/XAC1900/XAC1931/XAC1888/XAC1904/XAC1892/XAC1987/XAC3132/XAC1894/XAC1909/XAC1890 | 27 |
| **xac02020** | Two-component system | 33/70 | 146/1340 | 1.88e-15 | 2.82e-14 | 2.28e-14 | XAC0346/XAC2982/XAC2983/XAC1893/XAC1577/XAC4023/XAC1902/XAC1666/XAC1891/XAC1746/XAC2447/XAC1899/XAC0611/XAC1906/XAC1930/XAC1897/XAC1896/XAC1903/XAC1996/XAC2448/XAC1932/XAC1900/XAC1888/XAC1933/XAC1904/XAC1892/XAC1987/XAC1975/XAC3132/XAC1894/XAC1909/XAC1989/XAC1890 | 33 |
| **xac00920** | Sulfur metabolism | 12/70 | 31/1340 | 1.03e-08 | 1.03e-07 | 8.30e-08 | XAC3040/XAC0830/XAC3341/XAC3039/XAC0827/XAC0849/XAC0848/XAC0829/XAC0828/XAC3332/XAC3331/XAC3330 | 12 |
| **xac02040** | Flagellar assembly | 8/70 | 41/1340 | 0.0009 | 0.007 | 0.005 | XAC1908/XAC1988/XAC1974/XAC1933/XAC1973/XAC1975/XAC1909/XAC1989 | 8 |

**Supplementary Table 5. Bacterial strains and plasmids used in this study.**

| **Strain or plasmid** | | **Relevant characteristics** | **Reference** |  |  |
| --- | --- | --- | --- | --- | --- |
| **Strains** | |  |  |  |  |
| *Escherichia coli* |  | |  |  |  |
| DH5α | | F^–^ *rec*A (r_K_–, m_K_+) *hsd*R17 (rΔ(*lac*ZYA-*arg*F) Φ80d*lac*Z ΔM15 | New England Biolabs | |  |
| *Xanthomonas citri* pv*. citri* 306 | |  |  |  |  |
| *X. citri* | | Wild type Ap^r^ |  |  |  |
| *ΔfliC* | | In frame deletions of *fliC* | (9) |  |  |
| Δ*pilT* | | In frame deletions of *pilT* | This study |  |  |
| *ΔgumD* | | In frame deletions of *gumD* | This study |  |  |
| *ΔXAC2868* | | In frame deletions of *XAC2868 (vieA)* | This study |  |  |
| *ΔXAC2870* | | In frame deletions of *XAC2870 (cheB)* | This study |  |  |
| **Plasmids** | |  |  |  |  |
| pNTPS138 | | Suicide vector for generation of gene knockouts, *sacB* and Km^r^ | (10) |  |  |
| pNPTS-*pilT* | | pNPTS138 derivative for generation of *pilT* knockout, *sacB* and Km^r^ | This study |  |  |
| pNPTS-*gumD* | | pNPTS138 derivative for generation of *gumD* knockout, *sacB* and Km^r^ | This study |  |  |
| pNPTS-XAC2868 | | pNPTS138 derivative for generation of *XAC2868* knockout, *sacB* and Km^r^ | This study |  |  |
| pNPTS-XAC2870 | | pNPTS138 derivative for generation of *XAC2870* knockout, *sacB* and Km^r^ | This study |  |  |

       Km^r^, kanamycin-resistant; Ap^r^, ampicillin-resistant.

**Supplementary Table 6.** Summary of RNA-seq data of *X. citri* 306 grown in minimal medium XVM2m containing different carbohydrate sources or in LBON medium. Each sample represents a biologically independent experiment. QC: Quality Control.

| **Sample** | **Input reads** | **QC reads** | **QC reads (%)** | **rRNA reads (%)** | **Mapped reads (%)** |
| --- | --- | --- | --- | --- | --- |
| XVM2m_Glucose 1 | 2895851 | 2524189 | 87.17% | 5.64% | 87.68% |
| XVM2m_Glucose 2 | 5660364 | 4990317 | 88.16% | 1.92% | 91.82% |
| XVM2m_Glucose 3 | 5540215 | 5018731 | 90.59% | 2.58% | 68.56% |
| XVM2m_Glucose 4 | 5164471 | 4599580 | 89.06% | 0.79% | 69.50% |
| XVM2m_Glucose 5 | 4847539 | 4237597 | 87.42% | 14.36% | 61.53% |
| XVM2m_Glucose 6 | 23704254 | 19279704 | 81.33% | 22.76% | 73.24% |
| XVM2m_Cellobiose 1 | 5169619 | 4564215 | 88.29% | 6.83% | 86.44% |
| XVM2m_Cellobiose 2 | 3125511 | 2787587 | 89.19% | 30.06% | 88.34% |
| XVM2m_Cellobiose 3 | 3742334 | 3170705 | 84.73% | 0.91% | 87.39% |
| XVM2m_Cellobiose 4 | 6057031 | 5509092 | 90.95% | 33.62% | 84.61% |
| XVM2m_Starch 1 | 8486939 | 7186963 | 84.68% | 4.05% | 84.98% |
| XVM2m_Starch 2 | 4554717 | 3935401 | 86.40% | 3.17% | 95.30% |
| XVM2m_Starch 3 | 4102919 | 3392366 | 82.68% | 18.30% | 94.20% |
| XVM2m_Starch 4 | 5073033 | 4483765 | 88.38% | 22.24% | 82.07% |
| LBON 1 | 7182071 | 6247424 | 86.99% | 6.86% | 99.38% |
| LBON 2 | 5126895 | 4465913 | 87.11% | 8% | 99.56% |
| LBON 3 | 5641467 | 4955965 | 87.85% | 1.21% | 99.57% |
| LBON 4 | 16808541 | 14559558 | 86.62% | 69.77% | 98.53% |
| Medium input reads: | 122883771 |  | Medium mapped reads (%): | | 86.32% |

**Supplementary Table 7. Oligonucleotides used in this study.**

| **Name** | **Sequence (5’ to 3’)** |
| --- | --- |
| ***For mutant construction*** |  |
| **F1ΔpilT** | TCTAAGCTTCTGCCGGAACAGTGCCTGCAT |
| **R1ΔpilT** | GCTTGTCCTTGGCTTCAGCGATATCCATGC |
| **F2ΔpilT** | GCCAAGGACAAGCGGATATTCGAGTGATACG |
| **R2ΔpilT** | TGTAAGCTTACCAGCGGCGTGCCCAACATGA |
| **F1ΔgumD** | ATCAAGCTTGTCGAAGACTCGGAAAAGTCGC |
| **R1ΔgumD** | GTAGGTCGCGCTACTCAAGTCTGCCAAAAGCAT |
| **F2ΔgumD** | AGTAGCGCGACCTACTACTGATGATGGTGGGGAGCGT |
| **R2ΔgumD** | AGTAAGCTTGCATCGAATAAACGGTGAAGAACACG |
| **F1ΔXAC2868** | CATAAGCTTCAGCGTGGAACTGGCCGAAAGC |
| **R1ΔXAC2868** | GGCCAGCGTGCAGTTGCAGGAGAGAAG |
| **F2ΔXAC2868** | CAACTGCACGCTGGCCTGAGCCGATGAGCACCGCCA |
| **R2ΔXAC2868** | AGTAAGCTTCAGTGGCGATGACGTCGATGC |
| **F1ΔXAC2870** | CATAAGCTTCCTATCTGATGGCATCGCCGAT |
| **R1ΔXAC2870** | CACCACTGCCGAATCGTCGACCACCAT |
| **F2ΔXAC2870** | CGATTCGGCAGTGGTGATCGTGACGCAGCTGCAATAAC |
| **R2ΔXAC2870** | GAGTAAGCTTAGTATGTGGAGCGGCTCAAGCG |

**References**

1. Kanehisa M, Furumichi M, Tanabe M, Sato Y, Morishima K. 2017. KEGG: New perspectives on genomes, pathways, diseases and drugs. Nucleic Acids Res 45:D353–D361.

2. Consortium TU. 2021. UniProt: the universal protein knowledgebase in 2021. Nucleic Acids Res 49:D480–D489.

3. Lin Y, Liao YY, Huang RX, Li AZ, An SQ, Tang JL, Tang DJ. 2021. Extracellular amylase is required for full virulence and regulated by the global posttranscriptional regulator RsmA in *Xanthomonas campestris* pathovar *campestris*. Phytopathology 111:1104–1113.

4. Sato T, Hasegawa N, Saito J, Umezawa S, Honda Y, Kino K, Kirimura K. 2012. Purification, characterization, and gene identification of an α-glucosyl transfer enzyme, a novel type α-glucosidase from *Xanthomonas campestris* WU-9701. J Mol Catal B Enzym 80:20–27.

5. Watanabe R, Arimura Y, Ishii Y, Kirimura K. 2020. Crystal structure of α-glucosyl transfer enzyme XgtA from *Xanthomonas campestris* WU-9701. Biochem Biophys Res Commun 526:580–585.

6. Teufel F, Almagro Armenteros JJ, Johansen AR, Gíslason MH, Pihl SI, Tsirigos KD, Winther O, Brunak S, von Heijne G, Nielsen H. 2022. SignalP 6.0 predicts all five types of signal peptides using protein language models. Nat Biotechnol 40.

7. Krogh A, Larsson B, von Heijne G, Sonnhammer ELL. 2001. Predicting transmembrane protein topology with a hidden markov model: application to complete genomes. J Mol Biol 305:567–580.

8. Imai K, Asakawa N, Tsuji T, Akazawa F, Ino A, Sonoyama M, Mitaku S. 2008. SOSUI-GramN: high performance prediction for sub-cellular localization of proteins in Gram-negative bacteria. Bioinformation 2:417–421.

9. Andrade M de O, da Silva JC, Soprano AS, Shimo HM, Leme AFP, Benedetti CE. 2023. Suppression of citrus canker disease mediated by flagellin perception. Mol Plant Pathol 331–345.

10. Andrade MO, Farah CS, Wang N. 2014. The post-transcriptional regulator rsmA/csrA activates T3SS by stabilizing the 5′ UTR of hrpG, the master regulator of hrp/hrc genes, in *Xanthomonas.* PLoS Pathog 10.
